# Dr. Sims on the Subject of Inoculation for the Cow-Pox

**Published:** 1799-05

**Authors:** John Sims


### *To the Editors of the Medical and Phyfical Journal.*

IN laying before the public a cafe, which very accidentally fell into my hands, and in which the cow-pox was faid not to have proved a prefervative from the contagion of the fmall-pox, I had no intention of declaring myfelf an enemy to the inoculation of this difeafe; my only wifh was, to induce practitioners to paufe a little, to obtain more decided experience of its utility, before it fhould be *generally* recommended. It did not appear to me, that a fufficient number of trials had been then made, nor is the certainty that the having fuffered the vaccine difeafe, will prove a prefervative from the infection of the fmall-pox, as yet, lefs problematical than before. Much more experience is ftill wanting to warrant the *general* adoption of the meafure. I may then, I hope, be permitted to make a few obferva-tions, in explanation of what I have faid in the firft Number of the Medical Journal, without being fuppofed to engage in any controverfy. This is the more neceffary, as a very fenfible correfpondent, Mr. Laurence, feems to have mifunderftood, as well as to have mifquoted my words. It was far from my intention to affert, that " all hazardous experiments ought to be difcou-raged." I only intimate, that fuch rafh experiments, as the venturing to infert a variety of acrid animal poifons, by inoculation, into the human conftitution, ought to be difcouraged. In faying this, I certainly did not mean to throw any blame upon Dr. Jenner, for inoculating with the matter of the cow-pox. It was a received opinion in the country, that the having fuffered this difeafe, was a prefervative from the contagion of the human fmall-pox, and from fuitable inquiries made, Dr. Jenner found reafon to believe the fact. Now although all new experiments may be faid to be in fome degree hazardous, I am far from thinking that this, inftituted with a view to determine, whether the inoculated difeafe would have the fame effect, was one that ought not to have been made. On the contrary, I think every praife is due to Dr. Jenner, forfor bringing it to the fair teſt of experiment, and laying it before the public in the handſome manner he has done. Here was an object certainly worthy of the hazard that was to be run; but when Dr. Jenner, from having adopted the improbable opinion, that the diſeaſe owed its origin to the greaſe in horſes, ventured to inoculate with the matter of a putrid ſore, with a view of determining if this alſo had the power of preſerving from the contagion of the ſmall-pox, he was, I think, entering upon the field of raſh experiment. No immediate benefit was likely to accrue from ſuch a trial. It was indeed an inquiry of pathological curioſity; and had repeated experiments upon the cows already evinced, that this matter was capable of producing the diſeaſe upon the brute animal, I do not mean to ſay, that even this experiment, with due circumſpection, and a proper ſenſe of the hazard attending it, was in no caſe to have been made. But when I learn, that this and ſimilar experiments are making, in all the variety of modes, by different perſons, without any apparent fear of the poſſibility of doing any miſchief, it ſeems to me to be high time that the public ſhould know, that ſuch experiments may not only be attended with eminent hazard to the immediate ſubject of them, but that there is, at leaſt a *poſſibility*, that an infectious diſorder may thus be created. That the inoculation of ſome animal juices, in a certain ſtate of corruption, is capable of producing very dangerous diſeaſe in the individual, is too well known, from the accidents that have happened in proſecuting anatomical reſearches. That a diſeaſe of a contagious nature, can be thus excited, I do not aſſert, but if the thing be not in itſelf *abſolutely impoſſible*, the greateſt caution ought certainly to be uſed in inſtituting experiments from which there is only a diſtant *poſſibility* of ſuch a deplorable miſchief. For the ſake of illuſtration, let us ſuppoſe, that a writer upon the cow-pox, ſtarts the hypotheſis, that a certain febrile ſtate is excited in the ſyſtem, by the abſorption of the matter of the cow-pox, which, though not the ſame with the variolous fever, has nevertheleſs ſuch an analogy with it, that whoever has undergone this particular fever, will have the conſtitution ſo changed by it, that he ſhall be no longer liable to be affected by the contagion of the ſmall-pox;—another, wiſhing to bring this to the teſt of experiment, and expecting to throw light upon the pathology of the diſeaſe, inoculates ſeveral perſons with the matter of the pox of ſwine, or any other eruptive diſorder affecting animals, of any deſcription, merely to try if the fever excited by this matter will have the ſame effect, of enabling the conſtitution, ever after, to reſiſt the contagion of the ſmall-pox, I ſhould not heſitate to pronounce ſuch to be a raſh and hazardous experiment, not free from the *poſſible* danger of producing new diſeaſe.

The hiſtory of medicine informs us, that ſeveral diſeaſes have ariſen in the world, world, which were before unknown. The origin of these is certainly involved in great obscurity, but, as Dr. PEARSON observes, it has been often conjectured, that many of them are derived from brutes. Dr. GIRTANNER, in his very elaborate history of the syphilis, supposes this disease to owe its existence to the poison of a venemous insect, made use of by the natives of South America, for a lascivious purpose. And Dr. JENNER is himself inclined to suspect, that the small-pox may have been derived from the vaccine disease; perhaps he will consider the change which this disorder seems already to have undergone, in that it now frequently produces an eruption of pustules, in some cases not to be distinguished from the human small-pox, as a confirmation of this hypothesis. Indeed, if there has been no error * in the performing some of the operations, it should seem as if the two diseases are so nearly allied, that the one cannot be distinguished from the other. In one case that fell under my own observation, the disease produced by the inoculation for the cow-pox was accompanied by an universal eruption of pustules, which neither from their appearance, the accompanying fever, the time of the eruption, nor the period of their duration, could be any way distinguished from the distinct small-pox, yet this patient was inoculated under the care of a physician, (who has been very laudably employed in collecting such a mass of evidence, as must soon prove what advantage can be expected from substituting the cow-pox for the small-pox), with matter, as I was assured, taken by this gentleman himself, upon the point of a new lancet, from the arm of a child that had been previously inoculated with matter taken immediately from the cow.

If the same care was taken to use a new lancet for this first inoculation, there does not appear to be any other source of error, and as far as one case can be a proof, Dr. Jenner's suspicion of the origin of the small-pox, appears

* In these cases, where an eruption of pustules not to be distinguished from the small-pox, has been produced, it is difficult to avoid suspecting, that for want of due care, the same lancet may have been used for inoculating for the two diseases; by which means the small pox may have been communicated, where the cow-pox was intended. Perhaps, no ordinary washing will effectually deprive the lancet of the variolous infection. Many accidents have been said to happen of unintentional communication of the small pox, by letting blood with a lancet which had been long before used for the purpose of inoculation. Some time since, my father having inoculated a patient, and washed his lancet in warm water, was wiping it dry, when the patient accidentally received a slight wound in the finger. This wound was evidently infected with the poison, and went on in the same manner as the incision in the arm.

appears to be well founded *, as in this instance, where two persons only were inoculated in succession with the vaccine disease, a real small-pox, a disease in all its symptoms, its progress and termination, exactly similar to the human small-pox, is produced: of its infectious nature, indeed, I have no proof, although I have not the smallest doubt. Now let us suppose the human small-pox to be unknown in any country, where the vaccine disease prevails; a human subject is accidentally, or purposely, by way of experiment, inoculated with the poison from the teats of the cow, and others in succession are inoculated from the transferred disease, till a real small-pox is created, how many thousands would have to deplore the dreadful catastrophe of this unfortunate experiment. Who shall say, that if similar experiments were to be made with the poison of the pox of swine, or an eruptive disease of any other animal, a new contagious disorder might not be produced? But to hint at the possibility of such an event, is surely sufficient to prevent the undertaking of such rash and hazardous experiments.

*April 20th*, 1799 JOHN SIMS.

* It is only meant that this case, if no error attaches, goes to prove the identity of the two diseases; perhaps it may be thought more probable, that the vaccine disease is derived from the human, more especially as, in the brute, it appears to be a local disorder, affecting the same animal at different times.

